# Mésusage traditionnel du camphre: un danger oublié pour les enfants (à propos de 2 cas)

**DOI:** 10.11604/pamj.2019.32.89.17943

**Published:** 2019-02-26

**Authors:** Naoual Nchinech, Afaf Elgharbi, Fatima Zahra Aglili, Yamna Kriouile, Yahia Cherrah, Asmaa Aaloui Mdaghri, Samira Serragui

**Affiliations:** 1 Laboratoire de Pharmacologie et de Toxicologie, Faculté de Médecine et de Pharmacie, Université Mohammed V, Rabat, Maroc; 2 Pôle Pharmacie, Hôpital Militaire d’Instruction Mohammed V, Rabat, Maroc; 3 Centre Anti Poison et de Pharmacovigilance du Maroc, Rabat, Maroc; 4 Service Pédiatrie 2, Hôpital d’Enfants, Rabat, Maroc

**Keywords:** Mésusage, enfant, intoxication au camphre, Misuse, child, intoxication induced by camphor

## Abstract

Dans notre pays, le recours aux recettes de médecine traditionnelle et aux produits cosmétiques artisanaux est très fréquent en raison du taux élevé d'analphabétisme, du faible pouvoir d'achat et du grand nombre d'herboristes. Le camphre est un produit peu coûteux, facilement accessible et omniprésent dans presque toutes les maisons, le rendant potentiellement toxique en cas de mauvaise utilisation, en particulier chez les enfants. Nous rapportons ici l'histoire de 2 cas d'intoxication consécutifs à une recette de beauté à base de camphre en poudre. L'anamnèse donne des informations sur un empoisonnement par une poudre synthétique à base de camphre importé de Chine chez 2 enfants. Patiente 1: fille âgée de 2 mois, sans antécédents, admise aux urgences pédiatriques dans un état de pleurs incessants avec refus de manger. L'examen clinique est sans caractéristique particulière. Le test biologique standard était normal. Le nourrisson était sous surveillance neurologique, digestive et cutanée. Patiente 2: jeune fille de 6 ans admise à la suite d'une crise atonique avec syncope et mousse, suivie d'une douleur abdominale accompagnée d'une douleur abdominale accompagnée de vomissements alimentaires consécutifs à l'ingestion de lait. L'évolution était favorable après 48 heures de prise en charge symptomatique. L'entretien avec les mères a révélé qu'il s'agissait de deux voisins qui avaient reçu une recette traditionnelle pour le soin des cheveux d'un troisième voisin, après quoi ils avaient mélangé du camphre en poudre avec de l'huile d'olive, puis l'avaient appliquée pendant 1 heure sur les cheveux de leurs enfants, provoquant ainsi l'apparition de ces signes.

## Abstract (en)

In our country, the use of traditional medicinal recipes and artisan cosmetic products is very frequent due to the high rate of illiteracy, low purchasing power and of the large number of herbalists. Camphor is a low-cost product, easily accessible and omnipresent in almost every home, making it potentially toxic in case of misuse, in particular among children. We here report two cases of intoxication induced by beauty recipe made with powdered camphor. Patients’ medical history gave informations about poisoning in 2 children caused by synthetic powder made with camphor imported from China. Patient 1: little girl aged 2 months, with no previous medical history, admitted to the Emergency Department due to constant crying and refusal to eat. Clinical examination showed no abnormalities. Standard laboratory tests were normal. Neurological, digestive and cutaneous monitoring were performed. Patient 2: girl aged 6 years admitted with atonic seizure associated with syncope and foaming followed by abdominal pain and vomiting after ingesting milk. The evolution was favorable 48 hours after symptom management. Mothers reported two neighbors had received a traditional hair care recipe by a third neighbor. After that they had mixed powdered camphor with olive oil, then they had applied it to the hair of their children for one hour, thus causing the occurrence of these symptoms.

## Introduction

Mentionné dans le coran comme un aromatisant pour boissons, le camphre, nommé au Maroc *«al kafour»*, est une substance cristalline blanche à odeur pénétrante et goût piquant et aromatique, obtenue à l'origine par distillation de l'écorce du camphrier, *Cinnamomum camphora* [1]. C’est un composant majeur de nombreuses espèces de plantes aromatiques existant au Maroc, telles que *Rosmarinus officinalis*, *Salvia aucheri subsp*, *Blancoana et Artemisia herba-alba* [2]. Il est couramment utilisé depuis longtemps dans notre médecine traditionnelle en mélange à de l’huile d’olive pour masser les muscles endoloris et activer la circulation sanguine des jambes. Des herboristes marocains prétendent même qu’en inhalant les effluves l’espace d’une seconde, de temps à autre, cela dégage le nez et chasse les idées noires. Aujourd'hui il est produit synthétiquement à partir de pinène, un hydrocarbure dérivé de l'essence de térébenthine. Le camphre peut être utilisé comme antitussif, antimicrobien, antiviral et agent analgésique, ainsi qu'un insecticide et un activateur de pénétration cutanée. Il est présent dans de nombreux produits cosmétiques, et assainisseurs d’air [3]. En absence de textes réglementaires encadrant la vente, les cubes de camphre sont fortement importés au Maroc et dans de nombreux pays notamment la Chine, sans étiquetage approprié et sans mise en garde. La forme cubique fait que le camphre soit confondu avec des morceaux de sucre ce qui augmente le risque d’intoxication par ingestion chez les jeunes enfants. Suivant le degré de l’exposition, le camphre peut causer chez les enfants de nombreux symptômes allant d’une simple céphalée jusqu’aux convulsions et même la mort par arrêt respiratoire après ingestion, inhalation ou exposition cutanée [4]. Nous rapportons 2 cas d'intoxications infantiles à symptomatologies différentes, consécutifs à une recette traditionnelle de beauté à base de tablettes de camphre.

## Patient et observation

**Figure 1:**
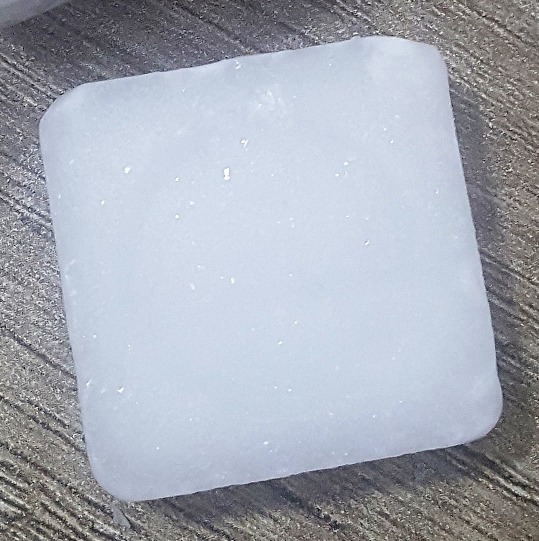
Tablette de camphre utilisée dans la recette de beauté causant l’intoxication

**Cas 1:** une petite fille de 2 mois, sans antécédents pathologiques, a été admise aux urgences pédiatriques dans un état de pleurs incessants. L’histoire de la naissance était normale et le mode d’allaitement était exclusivement maternel. La mère a indiqué qu’elle avait appliqué sur les cheveux de son enfant de la poudre de camphre obtenue en râpant puis en tamisant 2 tablettes de camphre de 7,08 gramme chacune, mélangées avec de l’huile d’olive. Le produit importé de Chine (Figure 1) est commercialisé sous forme de cube composé de quatre tablettes de résine de camphre, la teneur en camphre pur mentionnée étant de 99%. Le mélange a été posé pendant 1 heure sur les cheveux de l’enfant, engendrant des cris incessants avec refus de téter. L’examen somatique général été sans particularité. Elle a été ensuite hospitalisée, et prise en charge symptomatiquement. Sa mise sous surveillance neurologique, digestive et cutanée pendant 72 heures n’a révélé aucun épisode convulsif ou autre anomalie. Durant cette période, le Centre Antipoison et de Pharmacovigilance du Maroc a été sollicité pour établir une recherche des toxiques dans le sang par LC-DAD qui s’est avérée négative.

**Cas 2:** une jeune fille de 6 ans, a été admise aux urgences 2 heures après la survenue d’une crise atonique avec syncope et mousse aux lèvres. La reprise de conscience était accompagnée de douleurs abdominales et de vomissements alimentaires consécutifs à l’ingestion du lait motivant la consultation. Sa mère a révélé qu’elle avait appliqué le même mélange pendant une heure sur les cheveux de la petite, sauf qu’elle a utilisé 6 tablettes de camphre en plus. La fille avait comme antécédents une amygdalectomie, une adénoïdectomie à l’âge de 2 ans, une anémie ferriprive dont le traitement à été arrêté 4 ans avant et une notion de consanguinité 2^ème^degré. Elle a présenté une irritabilité à l’examen clinique sans autres signes associés. La recherche des toxiques dans le sang et l’urine était négative. L’enfant a été mis sous surveillance au niveau hospitalier pendant 3 jours au cours desquels une évolution favorable a été observée au bout de 2 jours de traitement symptomatique. Dans un entretien téléphonique pharmaceutique mené 3 mois après l’incident avec la mère, elle a déclaré que l’enfant n’a pas eu d’épisodes de crises épileptiques, mais que des maux de tête de type migraine se sont produits durant 2 semaines après la sortie de l’hôpital.

## Discussion

Outre ses usages traditionnels, le camphre est le plus fréquemment utilisé dans le traitement d'appoint à visée décongestionnante au cours des affections respiratoires banales (rhume, toux, bronchite simple) [5]. De ce fait, bien qu’il soit utilisé notamment par les herboristes, il est aussi prescrit par les cliniciens et peut être obtenu sans ordonnance. Peu de données existent sur la toxicité et la mortalité due au camphre au Maroc même s’il est facilement accessible. La littérature rapporte que le camphre en petite quantité peut causer une toxicité grave et même tuer l'enfant [6]. L'entretien pharmaceutique avec les mères a révélé qu'il s'agissait de deux voisines qui avaient reçu une recette traditionnelle pour le soin des cheveux d'une troisième voisine, après quoi ils avaient mélangé de la poudre de camphre avec de l'huile d'olive, puis l'avaient appliquée pendant 1 heure sur les cheveux de leurs enfants, provoquant ainsi l'apparition des signes sus-cités. La structure terpénique cyclique du camphre le rend hautement lipophile, expliquant à la fois son mouvement rapide à travers les muqueuses et le grand volume de distribution [7]. Chez le nourrisson et le jeune enfant, le passage cutané des substances appliquées sur la peau est plus important que chez l’adulte en raison d’un rapport surface corporelle/poids plus grand ou encore d’une hydratation plus importante de la couche cornée.

En outre, la barrière hémato-encéphalique chez l’enfant est plus perméable aux substances lipophiles, d’où un risque accru d’effets indésirables neurologiques en cas d’exposition à un produit fortement lipophile comme le camphre [8]. Cette perméabilité est accentuée par l’adjonction de l’huile d’olive. Le camphre induit une sensation de chaleur et peut être à l’origine de nausées, hallucinations visuelles, délire, œdème cérébral, état de mal épileptique, hypotension, tachycardie et insuffisance respiratoire [9]. La toxicité clinique disparaît généralement en 24 heures [7]. La majorité de patients ne sont pas suivis après la sortie de l’hôpital, de ce fait, l'incidence des effets toxiques à long terme du camphre est mal connue. Les cas décrits dans la littérature survenaient exclusivement après ingestion [10]. En 1988, Kôppel C *et al.* a signalé le premier cas de persistance de déficits neurologiques après ingestion de camphre. Dans la présente étude, des maux de tête de types migraine ont été rapportés en post-hospitalisation avec la 2^ème^ patiente.

Un entretien pharmaceutique de sortie de 30 minutes a été accordé aux mères pour expliquer les dangers liés à l'utilisation anarchique du camphre et des recettes traditionnelles. L'entretien avec les mères a révélé que 3 autres personnes utilisaient cette préparation pour leurs enfants, sauf que la durée d'exposition était inférieure à 30 minutes, ce qui pourrait justifier l'absence de symptômes néfastes. Dès lors, un module sur les préparations traditionnelles a été intégré aux entretiens pharmaceutiques avec les mamans des enfants hospitalisés afin de les sensibiliser sur les dangers encourus.

## Conclusion

Dans des pays comme le Maroc où le camphre est facilement accessible et très utilisé, l’éducation des parents concernant la toxicité potentielle du camphre et le danger qu’il représente pour leurs enfants, en cas d’utilisation dans des recettes traditionnelles, constitue une part importante de la prévention de l'intoxication. Une législation stricte appropriée devrait être mise en place pour assurer la vente réglementée du camphre avec un étiquetage approprié et une mise en garde. L’éducation des professionnels de santé exerçant dans le domaine de pédiatrie quant à la gestion d'un cas d'empoisonnement au camphre est importante. En effet, il doit toujours considérer l'intoxication au camphre comme une cause éventuelle d'apparition soudaine de crises convulsives chez l’enfant.

## Conflits d’intérêts

Les auteurs ne déclarent aucun conflits d’intérêts.
